# A Case Study on Integrating a New Key Event Into an Existing Adverse Outcome Pathway on Oxidative DNA Damage: Challenges and Approaches in a Data-Rich Area

**DOI:** 10.3389/ftox.2022.827328

**Published:** 2022-04-28

**Authors:** Elizabeth Huliganga, Francesco Marchetti, Jason M. O’Brien, Vinita Chauhan, Carole L. Yauk

**Affiliations:** ^1^ Department of Biology, University of Ottawa, Ottawa, ON, Canada; ^2^ Mechanistic Studies Division, Environmental Health Science and Research Bureau, Health Canada, Ottawa, ON, Canada; ^3^ Ecotoxicology and Wildlife Health Division, Environment and Climate Change Canada, Ottawa, ON, Canada; ^4^ Consumer and Clinical Radiation Protection Bureau, Health Canada, Ottawa, ON, Canada

**Keywords:** adverse outcome pathways, reactive oxygen species, DNA damage, genotoxicity, toxicology

## Abstract

Adverse outcome pathways (AOPs) synthesize toxicological information to convey and weigh evidence in an accessible format. AOPs are constructed in modules that include key events (KEs) and key event relationships (KERs). This modular structure facilitates AOP expansion and network development. AOP development requires finding relevant information to evaluate the weight of evidence supporting each KER. To do this, the use of transparent/reproducible search methods, such as systematic review (SR), have been proposed. Applying SR to AOP development in a data-rich area is difficult as SR requires screening each article returned from a search. Here we describe a case study to integrate a single new KE into an existing AOP. We explored the use of SR concepts and software to conduct a transparent and documented literature search to identify empirical data supporting the incorporation of a new KE, increase in cellular reactive oxygen species (ROS), upstream of an existing AOP: “Oxidative DNA Damage Leading to Chromosomal Aberrations and Mutations”. Connecting this KE to the AOP is supported by the development of five new KERs, the most important being the first adjacent KER (increase in ROS leading to oxidative DNA damage). We initially searched for evidence of all five KERs and screened 100 papers to develop a preliminary evidence map. After removing papers not containing relevant data based on our Population, Exposure, Comparator and Outcome statement, 39 articles supported one or more KERs; these primarily addressed temporal or dose concordance of the non-adjacent KERs with limited evidence supporting the first adjacent KER. We thus conducted a second focused set of searches using search terms for specific methodologies to measure these first two KEs. After screening, 12 articles were identified that contained quantitative evidence supporting the first adjacent KER. Given that integrating a new KE into an existing AOP requires the development of multiple KERs, this approach of building a preliminary evidence map, focusing evidence gathering on the first adjacent KER, and applying reproducible search strategies using specific methodologies for the first adjacent KER, enabled us to prioritize studies to support expansion of this data-rich AOP.

## 1 Introduction

### 1.1 Adverse Outcome Pathways: General Background and Challenges

Adverse Outcome Pathways (AOPs) synthesize toxicological information from various sources to convey and weigh evidence in an accessible and transparent format (Ankley et al., 2010; Sakuratani et al., 2018). The AOP format provides a framework for organizing mechanistic information that describes the chain of events from a molecular initiating event (MIE) through a series of intermediate key events (KEs) to an adverse outcome (AO) (Perkins et al., 2011). Key event relationships (KERs) explain the causal linkages between KEs and form the basis of using AOPs for predictive toxicology. By focusing on mechanistic information, AOPs are generally referred to as chemically agnostic. They can thus be applied broadly as a predictive tool for AOs induced by chemicals tested using high-throughput and high-content mechanistic data sources. They are also used to support test method development, identify knowledge gaps, and direct priority research.

A central premise of the AOP program is that AOPs are modular, with KE and KER units that can be used in multiple pathways, facilitating collaboration and the development of AOPs relevant to multiple stressors. This modular design also enables the expansion of existing AOPs and the creation of AOP networks that more thoroughly explain toxicological effects. Thus, a foundational principle is that the creation of duplicate or similar KEs is to be avoided. KEs, which are measurable biological events, should be developed such that they are broadly applicable while specifics, such as timescale (acute or chronic) or cell type, should be described in the KER or in the overall AOP. However, the AOP-Wiki is open for submissions and AOP authors have varying levels of familiarity with AOP conventions. Moreover, the AOP framework is relatively new and the standardization of AOPs is ongoing. The creation of many virtually identical KEs in the AOP-Wiki by many different authors represents a critical challenge at present.

KERs summarize the available empirical data and biological knowledge to describe the causal relationships between KEs using the modified Bradford-Hill (B-H) criteria. These criteria include: 1) biological plausibility (i.e., the relationship’s consistency to current accepted mechanistic knowledge); 2) empirical evidence of dose, temporal, and incidence concordance between the two KEs; and 3) essentiality of the KEs (i.e., evidence that an upstream KE must occur in order to observe a downstream KE) (Meek et al., 2014). A summary of the quantitative understanding of the relationship between the two KEs and consideration of uncertainties and inconsistencies is also provided in KER development. The overall weight of evidence of an AOP is supported by KERs that are both adjacent and non-adjacent (OECD, 2018); indeed, empirical evidence linking non-adjacent KEs in an AOP often provides some of the greatest support if intermediate KEs are less routinely measured.

To weigh the evidence supporting KERs and AOPs, AOP authors are required to summarize the current knowledge, find data meeting B-H criteria in support of the relationships, and identify uncertainties and inconsistencies within the literature. This evidence is often found across diverse sources and, for a well-studied research area, the amount of literature and data review required to identify evidence to support KERs and AOPs can be substantial. Moreover, empirical evidence and quantitative understanding are often difficult to mine from the literature. Given the extensive work required for AOP development in general, it has recently been proposed that KERs could be developed and reviewed independently, rather than the current process wherein entire AOPs are developed and reviewed (Svingen et al., 2021). These authors also propose that where possible pragmatic, transparent and documented methods, including the use of systematic review (SR) tools and approaches, be applied for KER and AOP development, as opposed to the narrative approach that is currently used in AOP literature review. They note, however, that this may not be practical for data-rich fields. The proposal by Svingen et al. (2021) is highly relevant to our interest to build on an existing series of AOPs in the area of genotoxicity assessment (described in more detail below).

Within AOP development there are no strict guidelines on how to gather literature evidence for a KER. However, as more institutions recognize the importance of SR for regulatory evaluations (Whaley and Halsall, 2016), it has been proposed that transparent search methods also be used to develop AOPs (Leist et al., 2017). Reproducible search methods transparently describe how literature was searched and reviewed when weighing the collected evidence. There are a variety of search methods applied today including scoping reviews, systematic evidence mapping (SEM), and SR; SR is the most widely used. SEMs provide a visual summary of systematically gathered research. They are often conducted to characterize gaps and summarize evidence with a broad scope and do not require risk-of-bias analysis before focusing a SR on a specific question (Wolffe et al., 2019). SR is a framework that aims to answer a specific research question by using reproducible search methods to synthesize all available data. A variety of SR tools are now available, including software tools to facilitate database creation, filtering, review, and prioritization. Using a transparent and documented approach to literature searches provides a stronger overall weight of evidence to the resulting AOP and KER components and informs on uncertainties and inconsistencies in the database.

Below we present a case study to investigate approaches for creating and integrating a single KE upstream of an existing AOP and applying SR tools to collect evidence supporting KERs to “network” into this existing AOP. We anticipated challenges in the literature search because this is an extremely data-rich area, and the work was to be done by a single researcher (SRs require a team). Therefore, the SR method of screening each article returned from a search was not feasible. Instead, SR concepts were used to develop a transparent and documented approach to literature review that could provide a strong overall weight of evidence and inform on uncertainties and inconsistencies without the screening of an excessive number of articles. A SR software tool was used to screen the collected articles with defined inclusion/exclusion criteria; then, an evidence map was built to visualize the evidence collected.

### 1.2 The Use of Adverse Outcome Pathways in Genotoxicity Assessment

Genotoxicity assessment determines the ability of chemicals to cause genetic damage, such as chromosomal aberrations and mutations, which can lead to cancer (Hoeijmakers, 2009) and genetic disease (Caldecott, 2008). Products in commerce are required to be assessed for their genotoxic potential in order to protect human and ecological health. At present, conventional tests typically classify chemicals as genotoxic or non-genotoxic but provide limited to no mechanistic information about how the test chemicals cause genotoxicity-associated AOs (Dearfield et al., 2017).

**FIGURE 1:**
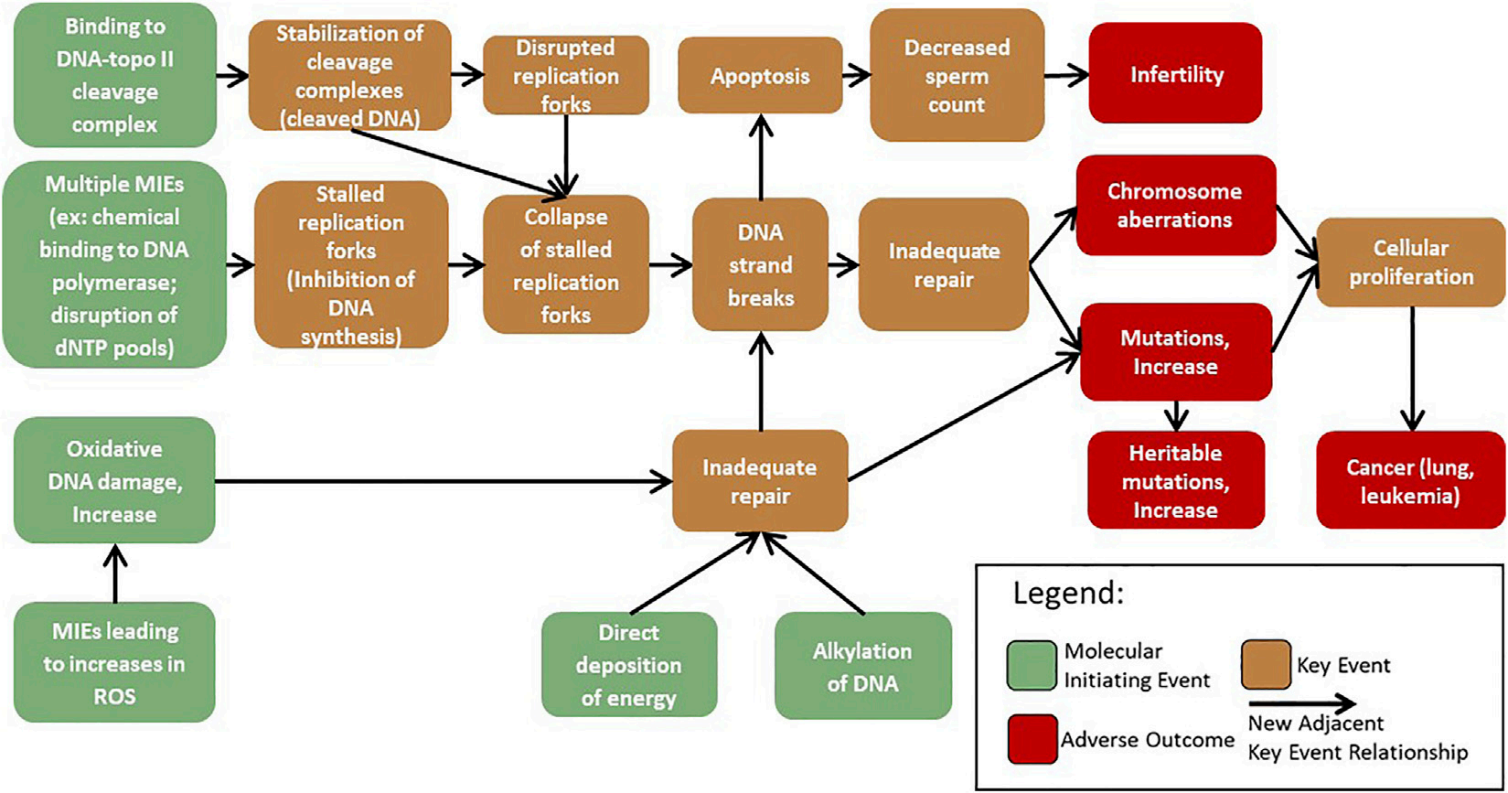
AOPs that converge on KEs associated with genotoxicity were extracted from the AOP-wiki and adapted from those proposed by Sasaki et al. (2020). These AOPs were chosen to demonstrate the growing network of shared KEs and KERs that form a network leading to chromosome damage and mutations. A network of KEs leading to aneuploidy is being developed separately. MIEs: molecular initiating events.

To facilitate the development of mechanism-based testing and its use in genotoxicity assessment, AOPs corresponding with mechanisms of genotoxicity are under development (Yauk et al., 2013; Sasaki et al., 2020). There is thus an expanding number of genotoxicity-focused AOPs (e.g., Chauhan et al., 2021, https://aopwiki.org/aops/272; Marchetti et al., 2016, https://aopwiki.org/aops/106; Yauk et al., 2015, https://aopwiki.org/aops/15) in various phases of development. At present, the AOP-Wiki (https://aopwiki.org/aops) includes three genotoxicity AOPs that use the KEs “inadequate repair”, “mutations”, or “chromosomal aberrations”. One of these AOPs terminates in permanent genomic damage in offspring, reflecting interest in the use of genotoxicity as an endpoint of regulatory concern (Heflich et al., 2020); whereas the other two AOPs lead to cancer outcomes, which are established endpoints of regulatory concern. One of these AOPs is endorsed by the Organisation for Economic Co-operation and Development (OECD), the other two are presently under internal review by the Extended Advisory Group on Molecular Screening and Toxicogenomics (EAGMST). Endorsement indicates that an AOP has been subject to external review, approved by EAGMST, and finally “endorsed” by the Working Group of the National Coordinators for the Test Guidelines Programme (WNT) and the Working Party on Hazard Assessment (WPHA). Endorsement by the WNT and WHPA indicates that the appropriate scientific review process was followed and the AOP is deemed to be worthy of public dissemination (OECD, 2021). Additional AOPs that will be merged with this genotoxicity network have been described by the Genetic Toxicology Technical Committee (GTTC) of the Health and Environmental Sciences Institute (HESI) and are in various stages of development (Sasaki et al., 2020). With the expanding genotoxicity AOP network (Figure 1) there is a need to further harness the modular nature of the AOP framework and collaborate to develop additional KEs applicable to multiple pathways that inform genotoxic mechanisms and outcomes.

### 1.3 Expansion of an Existing Adverse Outcome Pathway on Oxidative DNA Damage: This Project

**FIGURE 2:**
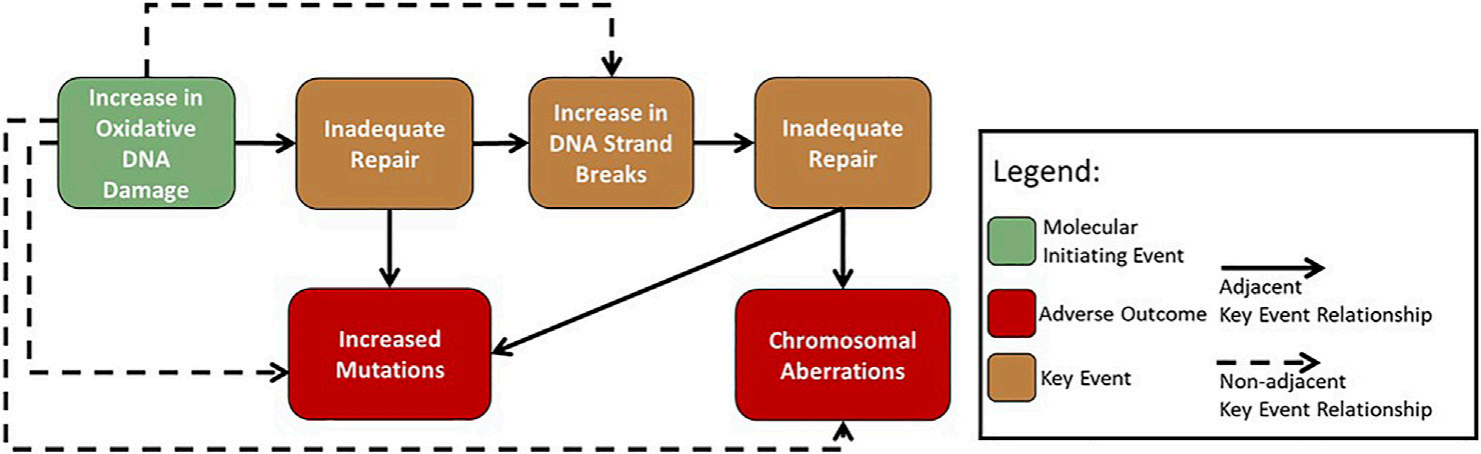
Flow diagram of the adverse outcome pathway ‘Oxidative damage leading to mutations and chromosomal aberrations’ (Cho et al., 2022).

Initial work within the GTTC developed AOP #296: ‘Oxidative DNA damage leading to chromosomal aberrations and mutations’ (Cho et al., 2022) (Figure 2). This pathway was first suggested during a GTTC workshop in 2017, where the pathway was used to demonstrate how AOPs could be harnessed to advance mode of action analysis in genetic toxicology (Sasaki et al., 2020). The AOP begins with an increase in oxidative damage to DNA (MIE) that overwhelms the repair capacity leading to inadequate repair of oxidative DNA damage (KE1). Inadequate repair of the oxidative DNA damage (KE1) then branches into two paths. It can lead to an increase in mutations (AO1), arising from replication of damaged template DNA, and/or to an increase in DNA strand breaks (KE2), occurring from the DNA repair process removing oxidative lesions. An increase in DNA strand breaks (KE2) can overwhelm the repair capacity and cause inadequate repair (KE1), leading to an increase in chromosomal aberrations (AO2) (Cho et al., 2022).

AOP #296 indicates that, in addition to direct oxidation of DNA by chemicals like hydrogen peroxide and other radicals, there are other upstream MIEs that indirectly cause oxidative DNA damage. Thus, the MIE for AOP #296 could also be considered an intermediate KE for other AOPs. Oxidative DNA damage includes a wide variety of DNA lesions such as modifications to, or removal of, nitrogenous bases, modifications to sugar components, and cross-linking of DNA with proteins (Birben et al., 2012). Oxidative DNA damage can be caused by exposure to a toxicant either directly by interaction between the toxicant and DNA, or indirectly through an increase in reactive oxygen species (ROS). Indeed, there are many direct and indirect mechanisms by which toxicants themselves or exposure to toxicants can produce ROS and subsequently lead to oxidative DNA damage.

The overarching aim of the present work is to build on AOP#296 through networking in an upstream KE reflecting increases in ROS and developing the relevant KERs for evaluation, aligned with the proposal of Svingen et al. (2021). The approach taken toward this objective was to: 1) identify the relevant new KE needed through a search of the AOP-Wiki; 2) determine if there are existing KERs that could be used to connect to AOP#296; and 3) apply freely available SR tools to facilitate collection of evidence to support the new KERs.

## 2 Methods

### 2.1 Key Event and Key Event Relationship Identification

We first aimed to identify a KE that describes increases in cellular levels of ROS. The AOP-wiki (https://aopwiki.org/) was searched using the terms “reactive oxygen species” and “oxidative stress” on the KE search page (https://aopwiki.org/events; using the “search key events” box) to identify existing KEs that might be suitable for incorporation in our pathway. Each KE was reviewed to examine the extent of development, its review status (e.g., part of an “endorsed” AOP), and whether KERs already exist to link the KE to other events in AOP#296.

### 2.2 Broad Literature Search

In this case study, there were two rounds of literature searches and screenings performed using a SR management tool. The first search was broad; the terms were chosen such that they encompassed all five KERs linking an increase in ROS to the KEs of AOP#296. The search was conducted to assess the available data and to determine more specific questions or data gaps. The second set of searches was conducted to address a specific KER that was underrepresented in the previous search.

The broad search was performed in the University of Ottawa online Library, using the OMNI search tool (ExLibris Primo, 2020). Two terms were chosen: “reactive oxygen species” to capture the proposed new KE increase in ROS and “DNA damage” to capture the KEs of AOP#296. The terms “reactive oxygen species” and “DNA damage” (see Table 1 for search syntax) were searched and filtered for journal articles available online. The 236,505 results were sorted by relevance according to OMNI’s Intelligent Ranking Technology. The Intelligent Ranking Technology ranks results based on word match, value score (query words in author, title, subject, plus date) and word proximity.

**TABLE 1:** Search strings for broad and focused literature searches.

Search Number	Search String	# of results retrieved (12 February 2021)
1 (broad search)	(Reactive Oxygen Species) AND (DNA Damage)	236,505

Focused searches		# of results retrieved (28 October 2021)
2	(FPG-modified comet) AND (Electron paramagnetic resonance spectroscopy)	2
3	(FPG-modified comet) AND (Electrochemical detection of ROS)	16
4	(FPG-modified comet) AND (HyPer)	4
5	(FPG-modified comet) AND (Hydroethidine)	0
6	(FPG-modified comet) AND (Mito-SOX)	1
7	(FPG-modified comet) AND (Mito-HE)	8
8	(FPG-modified comet) AND (Electrode detection of ROS)	9
9	(FPG-modified comet) AND (Boronate probes)	1
10	(FPG-modified comet) AND (CellROX)	0
11	(ELISA) AND (8-oxodG)AND (Electron paramagnetic resonance spectroscopy)	2
12	(ELISA) AND (8-oxodG) AND (Electrochemical detection of ROS)	73
13	(ELISA) AND (8-oxodG) AND (HyPer)	18
14	(ELISA) AND (8-oxodG) AND (Hydroethidine)	3
15	(ELISA) AND (8-oxodG) AND (Mito-SOX)	0
16	(ELISA) AND (8-oxodG) AND (Mito-HE)	11
17	(ELISA) AND (8-oxodG) AND (Electrode detection of ROS)	14
18	(ELISA) AND (8-oxodG) AND (Boronate probes)	1
19	(ELISA) AND (8-oxodG) AND (CellROX)	0
20	(LC-MS) AND (8-oxodG)AND (Electron paramagnetic resonance spectroscopy)	2
21	(LC-MS) AND (8-oxodG) AND (Electrochemical detection of ROS)	57
22	(LC-MS) AND (8-oxodG) AND (HyPer)	6
23	(LC-MS) AND (8-oxodG) AND (Hydroethidine)	1
24	(LC-MS) AND (8-oxodG) AND (Mito-SOX)	0
25	(LC-MS) AND (8-oxodG) AND (Mito-HE)	2
26	(LC-MS) AND (8-oxodG) AND (Electrode detection of ROS)	10
27	(LC-MS) AND (8-oxodG) AND (Boronate probes)	2
28	(LC-MS) AND (8-oxodG) AND (CellROX)	0
29	(HPLC-EC) AND (8-oxodG)AND (Electron paramagnetic resonance spectroscopy)	2
30	(HPLC-EC) AND (8-oxodG) AND (Electrochemical detection of ROS)	45
31	(HPLC-EC) AND (8-oxodG) AND (HyPer)	3
32	(HPLC-EC) AND (8-oxodG) AND (Hydroethidine)	0
33	(HPLC-EC) AND (8-oxodG) AND (Mito-SOX)	0
34	(HPLC-EC) AND (8-oxodG) AND (Mito-HE)	3
35	(HPLC-EC) AND (8-oxodG) AND (Electrode detection of ROS)	10
36	(HPLC-EC) AND (8-oxodG) AND (Boronate probes)	0
37	(HPLC-EC) AND (8-oxodG) AND (CellROX)	0

The 100 most “relevant” results as ranked by the software were chosen as the starting place to evaluate the types and quality of evidence available for this data-rich area. These results were uploaded to the SR management tool Covidence.org (Covidence, 2021) for screening and to track included and excluded articles at each stage. Covidence was also used to remove duplicates. The titles and abstracts of the remaining articles were screened using a modified Population, Exposure, Comparator and Outcome (PECO) statement. The standard PECO statement was modified to include Population, Exposure, and Endpoints (Table 2) to better fit the empirical evidence and quantitative understanding needed for our AOP. The population and exposure categories were broad, and the endpoints category included the KEs of our AOP: cellular ROS, oxidative DNA damage, inadequate repair, mutations, DNA strand breaks, and chromosomal aberrations. During the title and abstract screening, articles not meeting the modified PECO statement were excluded. After the title and abstract screening, the full texts of the remaining articles were screened for the measurement of cellular ROS, the measurement of one or more of the downstream KEs and for empirical evidence (dose, temporal, or incidence concordance between the measured KEs) or quantitative understanding (the inclusion of a control, or more than one dose or timepoint measured). During the full text screening, articles that did not measure cellular ROS, one or more downstream KE, and lacked empirical or quantitative evidence were excluded. Articles that passed screening were included as evidence for our proposed KERs, and data were extracted from these articles. The data extracted included the toxicant, model used, the relevant KEs, the assays used to measure the KEs, whether essentiality was measured, and the type of evidence that was contained (temporal, dose/concentration, incidence or conflicting).

**TABLE 2:** Population, Exposure, Endpoint statement, for the inclusion/exclusion of full text screening.

Modified PECO element	Evidence
Populations	Animal (all levels), human, *ex vivo*, *in vivo*, cell, cell-lines, epidemiological, cohort, population, organ, tissue, cellular, molecular, cellular components, biologically based models
Exposures	Reactive Oxygen Species (ROS), Reactive Nitrogen Species (RNS), Chemicals or Stressors causing an increased production of ROS/RNS (directly or indirectly)
Endpoints	All KEs in AOP: Cellular Reactive Oxygen Species (ROS), Oxidative DNA damage, Inadequate repair, Mutations, DNA strand breaks and Chromosomal aberrations

### 2.3 Specific Literature Search

Once the collected data from the broad search were assessed, a second focused search was conducted to specifically collect empirical data supporting the direct KER linking “increase in ROS” to ‘increase in oxidative DNA damage. The second search was performed on 28 October 2021, in the University of Ottawa online Library, using the OMNI search tool (ExLibris Primo, 2020). Terms (Table 3) were selected to represent reliable and quantitative assays for the measurement of cellular ROS and of oxidative DNA damage (see Table 1 for search syntax). Some selected methods for the measurement of oxidative DNA damage are not specific to the detection of oxidative DNA lesions and, therefore, the common oxidative lesion 8-oxodG was added to these searches. These terms were searched in all combinations, for a total of 36 searches. The titles and abstracts of these articles were then screened using a modified PECO statement. The modified PECO statement was the same as the broad search (Table 2), except the only used endpoints were cellular ROS and oxidative DNA damage.

**TABLE 3:** Quantitative and reliable methods measuring cellular ROS or oxidative DNA damage used in search terms for focused search.

Methods measuring cellular ROS	Methods measuring oxidative DNA damage
Electron paramagnetic resonance spectroscopy	FPG-modified comet
Electrochemical detection of ROS	ELISA AND 8-oxodG^a^
HyPer	LC-MS AND 8-oxodG^a^
Hydroethidine	HPLC-EC AND 8-oxodG^a^
Mito-SOX	—
Mito-HE	—
Electrode detection of ROS	—
Boronate probes	—
CellROX	—

a 8-oxodG was added to indicated search terms because these methods are not specific to the detection of oxidative DNA damage.

## 3 Results

### 3.1 Identification of Relevant Key Events and Key Event Relationships

A search of the AOP-Wiki revealed that many KEs addressing alterations in ROS already existed. There were sixteen potential KEs found in the AOP-Wiki search, with names spanning “reactive oxygen species”, “reactive oxygen species production”, “reactive oxygen and nitrogen species”, “mitochondrial reactive oxygen species”, and “oxidative stress”. These KEs are separately included in 60 AOPs as either MIEs or intermediate KEs, and their corresponding AOP-wiki pages are in various stages of development, ranging from having all fields completely blank, having most fields blank, to having all fields filled out. There were no existing KERs that contained content to link these KEs to oxidative DNA damage, mutations and chromosomal aberrations for AOP#296 or other AOPs.

**FIGURE 3:**
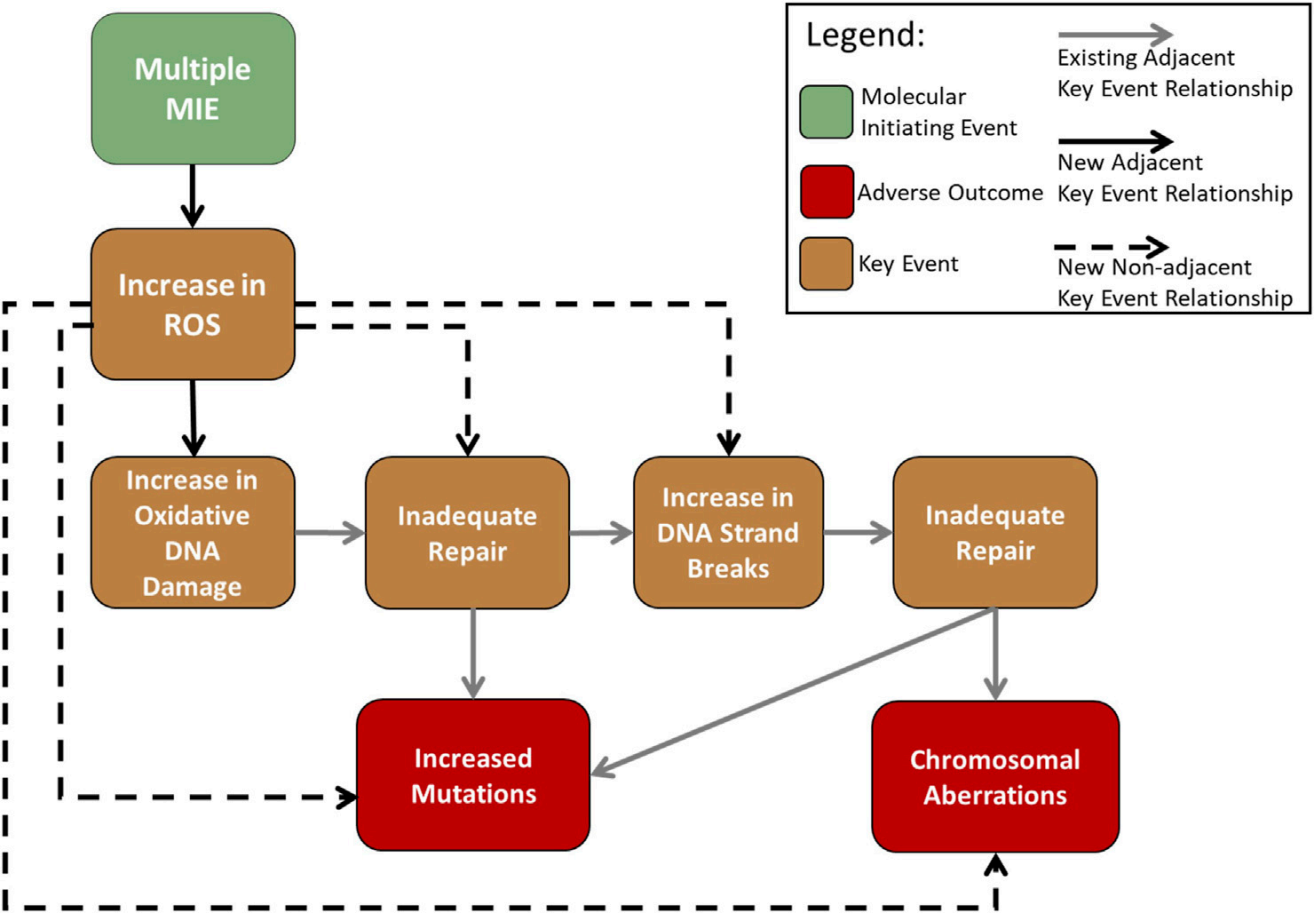
Flow diagram of the adverse outcome pathway “Oxidative damage leading to mutations and chromosomal aberrations” (Cho et al., 2022) with the inclusion of an upstream KE “increase in cellular ROS”, which represents the eventual consensus ROS KE, occurring after the multiple MIEs by which ROS are produced after toxicant exposure, and the resulting new KERs.

Having recognized redundancy of KEs in the AOP-Wiki as a general challenge for AOP development that needs to be addressed, a group of AOP “gardeners” (AOP framework specialists that have been recruited by the OECD to curate the information in the AOP-Wiki) is working on eliminating blank or unused KE. Because cellular ROS alteration is central to many toxicological pathways, an expert working group including these gardeners within the OECD has been created to develop a hub of consolidated KEs for this area that can be used by the broader community. The formation of this expert Working Group occurred following the launch of the present study. Thus, given our knowledge of, and participation in, this exercise, we decided to focus our work on the development of the new KERs that would be required to link the consensus ROS KE(s) upstream of AOP #296 MIE, “increase in oxidative DNA damage” (Figure 3). We determined that this would require investigating and potentially developing five new KERs: one adjacent KER—increases in ROS leading to oxidative DNA damage; and four non-adjacent KERs—ROS leading to inadequate DNA repair, ROS leading to DNA strand breaks, ROS leading to mutations, and ROS leading to chromosomal aberrations.

### 3.2 Broad Literature Search Results

**FIGURE 4:**
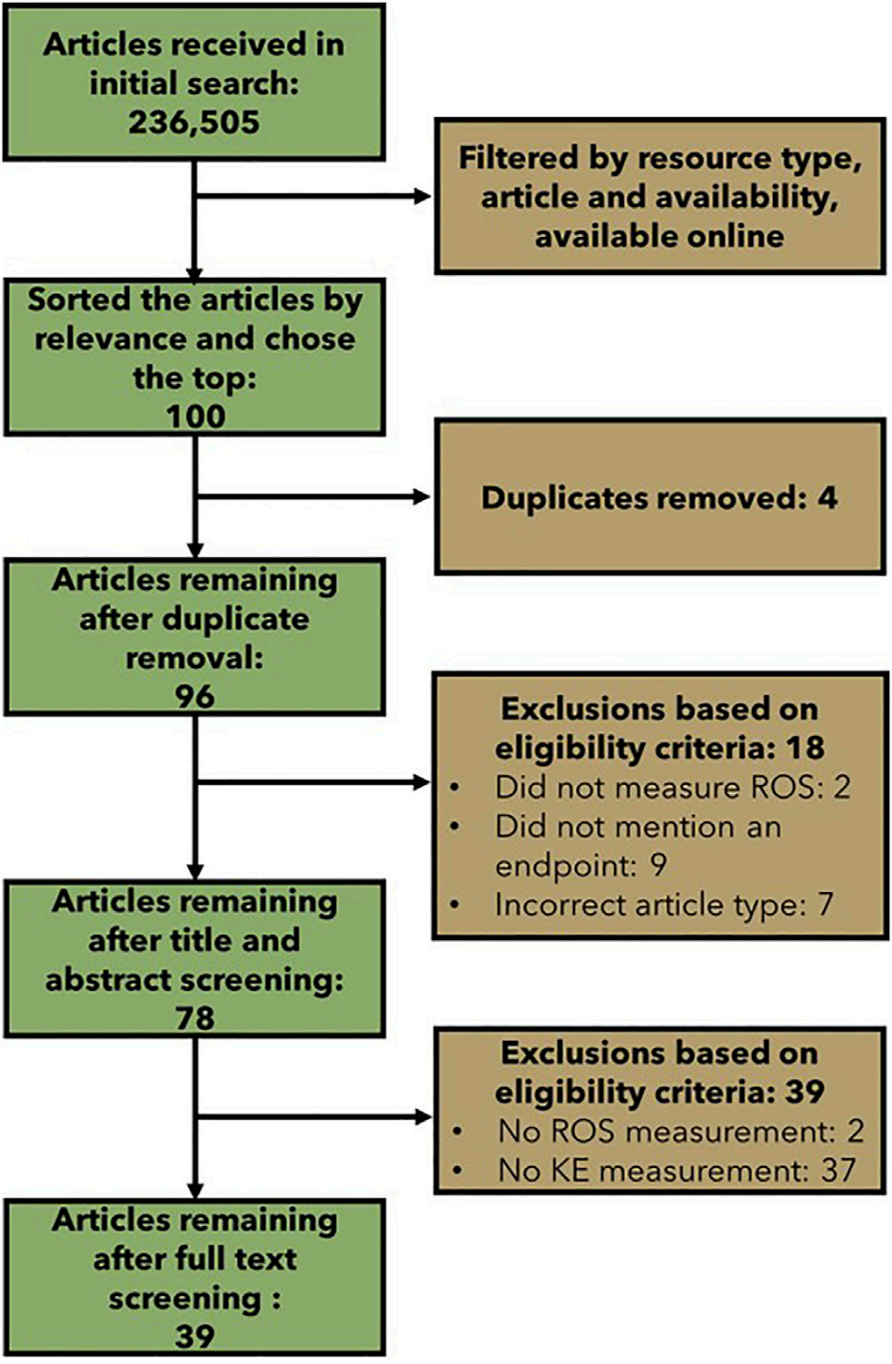
Flow chart showing the number of articles included and excluded at each step of the broad literature review.

The initial broad literature search led to the retrieval of 236,505 records, which is clearly beyond scope or scale of what is feasible and necessary to screen. We thus used a screening strategy to explore the top 100 articles. From these 100 papers, duplicate removal, title and abstract screening, and full text screening led to the elimination of 61 articles (Figure 4). Specifically, from the 100 most “relevant” results that were uploaded to COVIDence.org for screening, 4 duplicates were removed. The titles and abstracts of the remaining 96 articles were screened using a modified PECO statement (Table 2); 18 articles were excluded, and 78 articles remained for full text screening. The full texts of the remaining 78 articles were screened for the measurement of cellular ROS, the measurement of one or more of the downstream KEs, and the availability of empirical evidence (dose, temporal, or incidence concordance between the measured KEs) or quantitative data (the inclusion of a control, or more than one dose or timepoint measured). This resulted in the exclusion of 39 articles. The data were extracted from the remaining 39 articles.

The articles were tagged as to which KEs they measured and which KERs they supported to develop a “preliminary” evidence map (Table 4). As it was part of the selection criteria, all of the selected articles measured cellular ROS. The overwhelming majority of the articles also measured DNA strand breaks; 35/39 articles. Within these 35 articles, four also measured oxidative DNA damage, one measured inadequate repair and one measured mutations. The next most measured KE was oxidative DNA damage with 5 out of 39 articles. Within these five articles measuring oxidative DNA damage, four also measured DNA strand breaks, one measured inadequate repair, and one measured mutations. The remaining three KEs, inadequate repair, mutations, and chromosomal aberrations were measured in three, two, and one of the selected articles, respectively.

**TABLE 4:** Evidence map describing the number of articles identified through the screening that measured each key event (KE) in the “Oxidative DNA damage leading to chromosomal aberrations and mutations” pathway as well as increases in ROS.

	KE1: Oxidative DNA damage	KE2: Inadequate repair	KE3: DNA strand breaks	AO1: Mutations	AO2: Chromosomal aberrations
KE1: Oxidative DNA damage	5	1	4	1	0
KE2: Inadequate repair	—	3	1	1	1
KE3: DNA strand breaks	—	—	35	1	0
AO1: Mutations	—	—	—	2	1
AO2: Chromosomal aberrations	—	—	—	—	1

The articles were also tagged according to which of the B-H criteria empirical evidence types they supported (Table 5). Evidence supporting temporal concordance was found in 32 articles, 16 of which also addressed dose concordance. Dose concordance evidence was identified in 21 articles, 16 of which also provided evidence of temporal concordance. Incidence concordance evidence was not found in any articles. There was conflicting evidence of dose concordance in one article.

**TABLE 5:** Evidence map describing the number of articles retrieved in the screening that addresses each type of empirical evidence required to evaluate the weight of evidence of a KER.

	Temporal concordance	Dose/concentration concordance	Incidence concordance	Conflicting
Temporal concordance	32	16	0	0
Dose/concentration concordance	—	21	0	0
Incidence concordance	—	—	0	0
Conflicting	—	—	—	1

#### 3.2.1 Adjacent Key Event Relationships: Reactive Oxygen Species Leading to Oxidative DNA Damage

There were five papers that contained quantitative and empirical evidence of the relationship between an increase in ROS and an increase in oxidative DNA damage (Supplementary Table S1). Two of these papers measured endpoints that could be used to demonstrate that an increase in ROS occurs at lower or equal concentrations of the tested toxicants to those concentrations that induce an increase in oxidative DNA damage (Jacobsen et al., 2008; De Iuliis et al., 2009). One of the papers presents conflicting evidence; the endpoints measured show an increase in ROS at a higher concentration than an increase in oxidative DNA damage (Mittal and Pandey, 2014). Such types of conflicting data can occur because of differences in the dynamic ranges and sensitivity of the different methodologies used. Conflicting data could also occur because there is a certain increase in the level of ROS that would not be detected as a cell is able to control the ROS using antioxidants; an increase in ROS would only be detected once the cell is overwhelmed and cellular antioxidants are unable to control the ROS. Three of these papers provide empirical data to show that an increase in ROS occurs at earlier or the same timepoints to those timepoints that induce an increase in oxidative DNA damage (Babbar and Casero, 2006; Beattie et al., 2013; Meek et al., 2014).

#### 3.2.2 Nonadjacent Key Event Relationships

The empirical evidence collected for the non-adjacent KERs was enriched in papers supporting the relationship between an increase in ROS and an increase in DNA strand breaks. Indeed, there were 35 papers providing empirical evidence supporting an increase in ROS leading to DNA strand breaks (Supplementary Table S2), while there were only three papers supporting an increase in ROS leading to inadequate repair (Supplementary Table S3), two linking cellular ROS to mutations (Supplementary Table S4), and one linking cellular ROS to chromosomal aberrations (Supplementary Table S5). Within the 35 papers that supported an increase in ROS and an increase in DNA strand breaks, there were 30 papers demonstrating temporal concordance and 21 demonstrating dose/concentration concordance. Of the three papers that supported an increase in ROS leading to inadequate repair, all three addressed temporal concordance (Preston et al., 2009; Flaherty et al., 2017; Shih et al., 2021) and two provided data for dose/concentration concordance (Preston et al., 2009; Flaherty et al., 2017). There were no instances of incidence concordance or conflicting evidence. The two papers that supported an increase in ROS leading to mutations provided empirical evidence of temporal concordance (Jacobsen et al., 2008; Shih et al., 2021), while only one (Jacobsen et al., 2008) supported dose/concentration concordance. Finally, one paper that supported an increase in ROS leading to chromosomal aberrations showed temporal concordance (Shih et al., 2021).

### 3.3 Specific Literature Search Results

**FIGURE 5:**
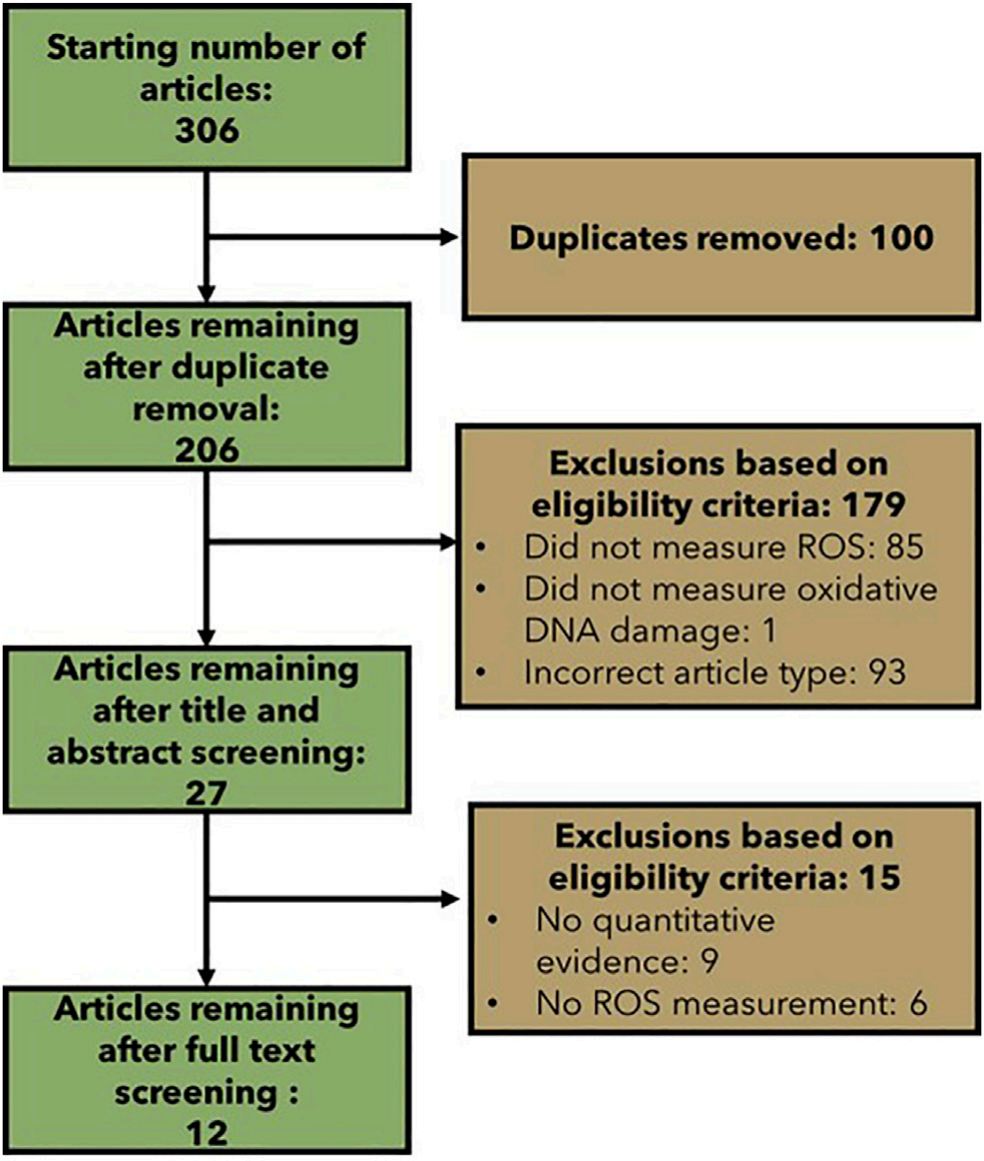
Flow chart showing the number of articles included and excluded at each step of the specific literature search.

The specific literature searches resulted in a total of 306 retrieved articles (Figure 5). 100 articles were duplicates across the searches. After title and abstract screening, 27 articles were identified that measured ROS and oxidative DNA damage and were eligible according to the modified Population, Exposure, Comparator and Outcome (PECO) statement (Table 2). After full text screening and data extraction, 12 articles were left that provided support for the quantitative relationship between ROS and oxidative DNA damage (Supplementary Table S6). The only searched method for oxidative DNA damage measurement that did not return a paper in the final data extraction was LCMS. Out of the 12 collected articles: four measured oxidative DNA damage with the Fpg-modified comet assay, four measured oxidative DNA damage with an ELISA and four measured oxidative DNA damage with HPLC. The methods for ROS measurement were varied. Three articles measured ROS with the oxidation of homovanillic acid, two articles measured ROS with HPLC, and one article measured ROS with each of the following methods: MitoSox fluorescence assay, chemiluminescence, glutathione-S transferase (GST) activity, mitochondrial matrix localized oxidant-sensitive ratiometric probe (mito-Ro-GFP), luminol-amplified chemiluminescence (LAC), HPLC and glutathione peroxidase (GPx) activity.

## 4 Discussion

Our case study explored the integration of a single KE into an existing AOP in a data-rich area. An initial challenge encountered was the existence of multiple KEs in the AOP-Wiki related to increases in cellular ROS that contained similar information. As KEs are purposefully modular to readily enable the development of AOP networks, this represents a significant challenge. Rather than choosing or developing the appropriate KEs, we decided to focus instead on KER development because a parallel activity to address redundancy in KEs is already underway within the OECD. Given that we identified five KERs that would be useful to bridge increases in ROS into AOP #296, we invested efforts in identifying an efficient, transparent and reproducible search strategy to collect relevant empirical evidence to support these KERs.

Our initial broad search retrieved over 200,000 articles, emphasizing the scale of research already conducted in this area and the extent of biological knowledge. It was clearly not feasible to screen and review these studies. Nevertheless, this initial broad search suggested that a SR may be beyond scope for our purposes (even narrowing by prototypical stressors), as suggested for data-rich AOPs by Svingen et al. (2021). Literature review tools were then used to retrieve and review the top 100 most ‘relevant’ articles meeting stringent B-H criteria. This provided the basis to begin to build a small database for this data-rich area that could be used to support the KERs and identify those with the most data even within this small pilot study. Indeed, from the 39 articles that made it through the screening process, our evidence map suggests there is extensive empirical evidence (35 articles; 90% of articles retrieved) for the relationship between an increase in ROS leading to DNA strand breaks. This also suggests that this is likely one of the strongest KERs supporting the overall weight of evidence that an increase in ROS can lead to mutations and chromosomal aberrations. However, while empirical evidence aligned with the B-H criteria was identified within these top 39 articles, there were limited data to develop a quantitative understanding linking the extent to which ROS levels increase prior to the manifestation of downstream KEs. This is a typical challenge identified by AOP authors, where quantitative understanding is generally one of the main knowledge/data gaps.

The KERs describing an increase in ROS leading to oxidative DNA damage (the adjacent KER) and inadequate repair (non-adjacent KER) were also insufficiently supported based on this preliminary screen. However, detailed literature reviews exist that link these events, providing strong biological plausibility of the relationships between ROS, oxidative DNA damage, and inadequate repair relationships. Thus, an extensive database of empirical evidence for these KERs is likely not necessary, as biological plausibility is considered the most important evidence criterion and is given a very strong weight (OECD, 2018).

A critical goal in AOP development is the collection of quantitative evidence for KERs to enable the development of predictive models. There is a growing body of literature demonstrating methods to build, and the potential use of, quantitative AOPs (Wittwehr et al., 2017; Foran et al., 2019; Perkins et al., 2019). One aim of our literature review was to identify quantitative evidence to determine threshold levels of ROS that must be surpassed to cause oxidative DNA damage and resulting genotoxicity. Unfortunately, we did not find strong data to support the quantitative relationships of the KERs within the 39 papers retrieved. There was some evidence of a quantitative dose or temporal response for all of the KERs, which could be developed into a quantitative understanding (e.g., linking ROS to oxidative DNA damage and ROS to DNA strand breaks). However, the amount of ROS measured in these papers was reported in different ways: relative fluorescence units (RFUs), units/hour, percent positive cells, and percent ROS generation of control. This results in the inability to translate the amount of ROS in one study to the amount of ROS in another study. Critically for this area, the role of endogenously produced ROS varies between cell types, cell culture media, and cellular environments (Milkovic et al., 2019); therefore, the ability to scavenge ROS will vary depending on the protocol and the cell type (Ray et al., 2012). Also, there was very little overlap in the cells or cell lines that were used, limiting the comparison of ROS production and scavenging ability between cell types. Most of the collected papers (30/39) used dichlorofluorescein diacetate (DCFH-DA) fluorescence to measure ROS. However, DCFH-DA cannot be reliably used to measure intracellular H_2_O_2_, as it tends to autofluoresce and to leak out of cells when deacetylated by endogenous deacetylases (Kalyanaraman et al., 2012). Since DCFH-DA is not suitable for the quantification of H_2_O_2_ or ROS molecules, and there are other more reliable fluorescent dyes for the quantification of ROS, the majority of the collected papers were not suitable for the purposes of developing quantitative understanding of the KERs.

Our preliminary mapping of the literature suggests that priority KERs for future literature searches or research would be those linking cellular ROS to chromosomal aberrations and mutations. The literature search found the least amount of evidence for these KERs; within the 39 papers, two provided evidence for ROS leading to mutations and one paper provided evidence for ROS leading to chromosomal aberrations. A secondary and tertiary screen could be implemented to review these gap areas if deemed necessary. For example, a tiered approach could be used where search terms are successively eliminated to focus in on the areas requiring further data (i.e., subsequent searches to focus exclusively on papers that measure mutations and chromosomal aberrations).

Finally, because we deemed the first adjacent KER to be the most important, and identified the need for quantitative evidence, we conducted specific searches to fill this gap. To do this we decided to do a set of 36 searches pairing each of what we determined were suitable methodologies to derive quantitative measures of the two KEs. These searches retrieved 12 articles following screening that measured a quantitative relationship between cellular ROS and oxidative DNA damage. The specific literature search was able to capture more empirical evidence as well as evidence of a quantitative understanding for the prioritized KER. The 12 papers retrieved provide evidence of the timing between events and the relationship between incidence/magnitude of increased ROS and oxidative DNA damage in different model systems. This evidence could be used in future studies to build predictive relationships.

### 4.1 Conclusions and Recommendations

As the OECD’s AOP program matures, an abundance of new AOPs are in development. A significant challenge is ensuring the re-use of existing KEs and KERs within the AOP-Wiki to facilitate both AOP development by reducing work and the creation of AOP-networks that provide a more thorough understanding of toxicological effects. Indeed, our initial challenge in this project was the existence of multiple KEs related to ROS and oxidative stress in the AOP-Wiki (16 KEs relating to ROS and oxidative stress that are included in 60 AOPs). We encourage authors to carefully review existing KE(R)s before creating new ones and for authors to remove KE(R)s from the AOP-Wiki that they have created but do not intend to develop.

A second challenge in AOP development is the need for more robust and transparent evidence collection to foster reproducibility and support the overall weight of evidence in evaluating AOPs. We explored concepts and tools in SR for this purpose to refine our search strategies. As non-SR-experts, we found this approach to be feasible and encourage further use in AOP development, as it provides a more transparent weight of evidence evaluation than the traditional narrative approach and more readily enables identification of inconsistencies in the literature. Our recommendations based on our experiences herein are as follows:

1) For data-rich areas, we propose that one approach might be a tiered literature review to produce evidence maps wherein the initial screen involves selecting a sub-set of the top papers (e.g., the most relevant), with search terms successively narrowed until sufficient evidence (as deemed by the team of authors) is collected. In the future, machine learning tools may also be deployed to facilitate use of SR tools for more efficient and documented literature searches in AOP development. Such tools already exist but were beyond the scope of our present work (e.g., Jornod et al., 2021).
2) In addition, filtering for specific, reliable assays that produce quantitative data could provide an alternative strategy to both reduce the collected articles and improve the likelihood of developing a quantitative understanding with the collected articles.
3) Other strategies to retrieve the most relevant articles that were not applied in this study and would be useful include filtering for specific well-studied stressors or for widely used models to narrow searches.

A third challenge is the extensive amount of time required to develop AOPs. Our work demonstrates that even the development of a single (or a small selection) of KERs can require a significant amount of work. Thus, the research herein supports providing the option of having the KER as a module for development. Allowing KER review, paired with developing more efficient and systematic approaches to evidence collection and review, should streamline AOP development and increase confidence in their application.

Overall, we found that the application of SR review concepts and tools to undertake more transparent and documented literature search methods was an improvement over narrative approaches in KER development. It will be important for the community of experts to recommend best practices that balance the strengths of SR tools in AOP development against the depth of analysis needed, in particular for data-rich areas where there is extensive biological understanding. In anticipation of achieving that broader consensus, we encourage dialogue between experts in SR tools and AOP developers to advance this area.

## Data Availability

The original contributions presented in the study are included in the article/Supplementary Material, further inquiries can be directed to the corresponding author.
