# Supplementary material for: A Case Study on Integrating a New Key Event Into an Existing Adverse Outcome Pathway on Oxidative DNA Damage: Challenges and Approaches in a Data-Rich Area
Source: Front Toxicol. 2022 Apr 28;4:827328. doi: 10.3389/ftox.2022.827328 (PMC9097222; doi:10.3389/ftox.2022.827328)
Supplement: Supplementary file 1 [file DataSheet1.PDF]

A case study on integrating a new key event into an existing adverse outcome pathway on oxidative DNA damage: challenges and approaches in a data-rich area

Elizabeth Huliganga, Francesco Marchetti, Jason M. O'Brien, Vinita Chauhan, Carole L. Yauk

Supplementary Materials

Table S1. Empirical evidence of the adjacent key event relationship between an increase in cellular ROS and oxidative DNA damage.

Citation	Toxicant	Model	ROS assay	Oxidative DNA damage assay	Evidence Dose/concentration	Evidence Temporal
(De Iuliis et al. 2009)	Radio-frequency electromagnetic radiation (RF-EMR)	human spermatozoa (*in vitro*)	DHE and MitoSOX Red (MSR) fluorescence assays	8-OHdG ELISA	**ROS:** Increased after exposure to 1.8 GHz RF-EMR at specific absorption rate (SAR) 1.0 W/kg for 16 h. **Oxidative DNA damage:** Increased after exposure to 1.8 GHz RF-EMR at specific absorption rate (SAR) 2.8 W/kg for 16 h	
(Jacobsen et al. 2008)	C60	MutaMouse lung epithelial cells (FE1-MML)	DCFH-DA fluorescence assay	Comet assay + FPG	**ROS:** Increased after exposure to 2.78 ug/ml C60 for 3 h. **Oxidative DNA damage:** Increased after exposure to 100 ug/ml C60 for 3 h.	
	SWCNT				**ROS:** Increased after exposure to 2.78 ug/ml SWCNTs for 3 h. **Oxidative DNA damage:** Increased after exposure to 100 ug/ml SWCNTs for 3 h.	
(Mittal and Pandey 2014)	Cerium oxide nanoparticles (CeO_2 NPs)	A549 (lung adenocarcinoma) cells	DCFH-DA fluorescence assay and cellular glutathione content	FPG modified comet assay	**ROS:** Increased after exposure to 10 ug/ml CeO_2 NPs for 6 h. **Oxidative DNA damage:** Increased after exposure to 1 ug/ml CeO_2 NPs for 6 h. **(Conflicting)**	**ROS:** Increased after exposure to 10 ug/ml CeO_2 NPs for 3 h. **Oxidative DNA damage:** Increased after exposure to 1 ug/ml CeO_2 NPs for 6 h.
(Babbar and Casero 2006)	Tumor necrosis factor alpha (TNF-a)	BEAS-2B (Human bronchial epithelial) cells	DCFH-DA fluorescence assay	8-OHdG ELISA		**ROS:** Increased after exposure to 10 ng/ml TNF-a for 30 min. **Oxidative DNA damage:** Increased after exposure to 10 ng/ml TNF-a for 30 min.
		HBEC3KT cells				**ROS:** Increased after exposure to 10 ng/ml TNF-a for 3 h.

						**Oxidative DNA damage:** Increased after exposure to 10 ng/ml TNF-a for 3 h.
(Beattie et al. 2013)	Luteinizing hormone (LH)	MA-10 Ledig cells	DCFH-DA fluorescence assay	Comet assay + BSO (L-Buthionine-sulfoximine)		**ROS:** Increased after exposure to 100 ng/ml LH for 5 min.<br>**Oxidative DNA damage:** Increased after exposure to 100 ng/ml LH for 2 h.

Table S2. Empirical evidence of the non-adjacent key event relationship between an increase in cellular ROS and DNA strand breaks.

Citation	Model	Toxicant	ROS assay	DNA strand break assay
(Pan et al. 2007)	HL-60 (human cancer)	t 5-hydroxy-3,6,7,8,3′,4′-hexamethoxyflavone (5-OH-HxMF)	DCFH-DA DHE and CMFDA fluorescence assays	DNA fragmentation
(Joseph et al. 2014)	Normal human Keratinocytes (HaCaT)	Gamma radiation	DCFH-DA fluorescence assay	Alkaline Comet assay
(Gagnaire et al. 2020)	Zebrafish (Danio rerio)	Tritium, a radioactive isotope of hydrogen	ROS stimulation index	Alkaline Comet assay
(Alsharif and Hassoun 2004)	Mice Peritoneal lavage cells (TCDD sensitive female C57BL/6J mice)	2,3,7,8-tetrachlorodibenzo-p-dioxin (TCDD) and Vitamin A or Vitamin E succinate	Reduction of cytochrome C assay	Alkaline elution technique
(Z. Wang et al. 2021)	Nitric acid donors nanocapsules (NO-NCPs)	pulsed laser irradiation	DCFH-DA and DHE fluorescence assays	Chromosomal fragmentation
(Gu et al. 2017)	Shrimp, *Litopenaeus vannamei* with and without LvPHB2 knockout	Vibrio alginolyticus infection	DCFH-DA fluorescence assay	Comet assay

(Russo et al. 2006)	Human spermatozoa	B(a)P	DCFH-DA fluorescence assay	Comet assay
(Jacobsen et al. 2008)	MutaMouse lung epithelial cells (FE1-MML)	Carbon black, C60 fullerenes or SWCNT	DCFH-DA fluorescence assay	Comet assay
(Beattie et al. 2013)	MA-10 Leydig cells	luteinizing hormone (LH)	DCFH-DA fluorescence assay	Comet assay
(Cai et al. 2007)	Bel-7402 cells (Liver Carcinoma)	$Cu(OP)_2$	DCFH-DA fluorescence assay	Comet assay
(Ren et al. 2016)	MCF-7 breast cancer cells.	Psoralidin (PSO)	DCFH-DA fluorescence assay	Comet assay
(Itziou et al. 2011)	Digestive Gland Cells and Haemocytes from land snails *Eobania vermiculata*	Heavy Metals cadmium, lead, or copper	DCFH-DA fluorescence assay	Comet assay
(Luukkonen et al. 2010)	SH-SY5Y neuroblastoma cells	FeCl2	DCFH-DA fluorescence assay	Comet assay (3 hours)
(Yin et al. 2019)	JB6 P+ (mouse epidermal cell line)	Arsenic and UVB radiation	DCFH-DA and DHE fluorescence assays	Comet assay (SSB), γ-H2AX immunofluorescent staining (DSB)
(Ni et al. 2014)	A549 (lung adenocarcinoma) cells	chrysophanol	DCFH-DA fluorescence assay	Comet assay and DAPI staining
(Gil et al. 2019)	A549 (lung adenocarcinoma) cells	2-Hydroxy-3′,5,5′-trimethoxychalcone (named DK-139)	DCFH-DA fluorescence assay	Comet assay and γH2AX (12h, 24h)

(Zhang et al. 2015)	Caco-2 cells (differentiated and undifferentiated)	Aflatoxin B1 (AFB1) and aflatoxin M1 (AFM1	DCFH-DA fluorescence assay	CometChip
(Li et al. 2010)	Chondrocytes from juvenile rabbit joint	ofloxacin	DCFH-DA fluorescence assay	CometChip
(Chan et al. 2017)	BEAS-2B (Human bronchial epithelial) cells	house dust mites (HDM)	CellROX and MitoSOX fluorescence assays, Nitrite assay and XO activity assay	CometChip, gamma-H2AX
(Pagoria et al. 2005)	Supercoiled DNA	CQ and CQ-related photosensitizers benzil, bezophenone, and 9-fluorenone (9-F)	TEMPO-9-AC fluorescence	conversion of supercoiled (phi)X-174 RF I double stranded DNA to open circular and linear forms.
(E. Wang et al. 2017)	Human sperm	Silver nanoparticles	DCFH-DA fluorescence assay and standard ROS production kits	DNA fragmentation, sperm chromatin dispersion method
(Weyemi et al. 2012)	Human non-tumoral thyroid cell line (HThy-ori3.1)	doxycycline forced expression of H-Ras	DCFH-DA fluorescence assay	gamma-H2AX immunofluorescence and double strand break kinetics in agarose gel

(Froeling et al. 2019)	MiaPaCa2 Rosa26 cells	Napabucasin (2-acetylfuro-1,4-naphthoquinone or BBI-608)	DCFH-DA fluorescence assay and GSH/GSSG-Glo assay (Promega)	phospho-histone gamma-H2AX
(Flaherty et al. 2017)	Breast Cancer Cell lines. TNBC: MDA-MB-231 and HCC38, non-TNBC: MCF-7 and MCF10a (TNBC - Triple negative breast cancer)	Stress hormones cortisol and norepinephrine (psychological stress)	Electrochemical sensors	phosphorylated γ-H2AX, Comet assay
(Brar et al. 2012)	A549 (lung adenocarcinoma) cells	bleomycin	Electron paramagnetic resonance (EPR)	qPCR (mtDNA and nDNA measured separately)
(Mittal and Pandey 2014)	A549 (lung adenocarcinoma) cells	Cerium oxide nanoparticles (CeO_2 NPs)	DCFH-DA fluorescence assay, measurement of cellular GSH level	standard Comet assay
(B. Lee et al. 2020)	*Escherichia coli*	Lactoferricin B like peptide (LBLP)	DCFH-DA fluorescence assay	TUNEL assay
(Nie et al. 2019)	Porcine early embryos	Thiamethoxam (TMX)	DCFH-DA and MitoSOX fluorescence assay	TUNEL assay and γH2AX
(De Iuliis et al. 2009)	Human spermatozoa (*in vitro*)	Electromagnetic radiation/Mobile phone radiation 1.8 GHz	DHE and MitoSOX Red (MSR) fluorescence assays	TUNEL Assay for measuring apoptotic DNA fragmentation

(Chen et al. 2020)	Mouse oocytes	Ionomycin	ROS Orange Working Solution fluorescence assay	γH2AX and DAPI staining
(K. Lee et al. 2015)	NT-407 cell, U2OS cells and Huh7 cells	Cyclo(phenylalanine-proline) (cFP)	MitoSOX and DCFH-DA fluorescence assays	γH2AX and Neutral comet assay
(Samara et al. 2021)	Mantle cell lymphoma (MCL) mature B-cell lymphoid neoplasm cells (Jeko-1, REC-1 and Z138 cells)	Deferasirox (DFX)	DCFH-DA fluorescence assay	γH2AX western blot
(Greenshields et al. 2017)	Female C57BL/6 mice injected with ID8 murine ovarian cancer cells, HEY1 and HEY2 ovarian cancer cells	Artesunate (ART)	DCFH-DA fluorescence assay	γH2AX western blot
(Ren et al. 2015)	MCF-7 breast cancer cells	Cucurbitacin B (Cuc B)	DCFH-DA fluorescence assay	γH2AX western blot
(Toduka et al. 2012)	(CHO)-K1 cells	ZnO, CuO, Fe3O4, TiO_2, and Ag nanoparticles	DCFH-DA fluorescence assay	γH2AX

Table S3. Empirical evidence of the non-adjacent key event relationship between an increase in cellular ROS and inadequate repair.

Citation	Toxicant	Model	ROS assay	Inadequate Repair assay	Evidence: Dose/concentration	Evidence: Temporal
(Shih et al. 2021)	Arecoline	OEC-M1 cell line (oral squamous cell carcinoma)	Flow cytometer ROS Assay Kit 520 nm (Invitrogen, Cat. 88-5930)	Host cell reactivation assay		**ROS:** Increased after exposure to 100 ug/ml arecoline for 4 h. **Inadequate Repair:** reduced repair capacity was observed after exposure to 100 ug/ml arecoline for 24 h.
(Flaherty et al. 2017)	Norepinephrine	MDA-MB231 (triple negative breast cancer (TNBC)) cells	Electrochemical sensors used to detect specific ROS/RNS species (H_2O_2, NO_2, $ONOO^-$ and NO	Comet assay	**ROS:** H_2O_2 was increased after 1 uM norepinephrine after 15 min. **Inadequate repair:** DNA strand breaks occurred after exposure to 1 uM norepinephrine for 30 min, after a 20 min repair period the DNA strand breaks were repaired	**ROS:** H2O_2 was increased after 1 uM norepinephrine after 15 min. **Inadequate repair:** DNA strand breaks occurred after exposure to 1 uM norepinephrine for 30 min, after a 20 min repair period the DNA strand breaks were repaired
	Cortisol				**ROS:** NO_2 was increased after 1 uM cortisol after 15 min. **Inadequate repair:** DNA strand breaks occurred after exposure to 1 uM cortisol for 30 min, after a 20 min repair period the DNA strand breaks remained	**ROS:** NO_2 was increased after 1 uM cortisol after 15 min. **Inadequate repair:** DNA strand breaks occurred after exposure to 1 uM cortisol for 30 min, after a 20 min repair period the DNA strand breaks remained
(Preston et al. 2009)	Cisplatin	HEK 293 (Human embryonic kidney) cells	DCFH-DA fluorescence assay	8-OHdG ELISA	**ROS:** Increased after exposure to 30 uM cisplatin for 1 h. **Inadequate repair:** Oxidative DNA lesions were increased after exposure to 30 µM cisplatin for 1 h and a recovery period of 30 min or 1 h post,	**ROS:** Increased after exposure to 30 uM cisplatin for 1 h. **Inadequate repair:** Oxidative DNA lesions were increased after exposure to 30 µM cisplatin for 1 h and a recovery period of 30 min or 1 h post, after a recovery period of

					after a recovery period of 0 h, 2 h, 3 h, and 4 h there was no increase in oxidative DNA damage.	0 h, 2 h, 3 h, and 4 h there was no increase in oxidative DNA damage.

Table S4. Empirical evidence of the non-adjacent key event relationship between an increase in cellular ROS and mutations.

Citation	Toxicant	Model	ROS assay	Mutation assay	Evidence: Dose/concentration	Evidence: Temporal
(Shih et al. 2021)	Arecoline	OEC-M1 cell line (oral squamous cell carcinoma)	Flow cytometer ROS Assay Kit 520 nm (Invitrogen, Cat. 88-5930)	Host cell reactivation assay		**ROS:** Increased after exposure to 100 ug/ml arecoline for 4 h. **Mutation:** Increased after exposure to 100 ug/ml arecoline for 24 h.
(Jacobsen et al. 2008)	C60 or SWCNT	MutaMouse lung epithelial cells (FE1-MML)	DCFH-DA fluoresence assay	cII mutation frequency	**ROS:** Increased after exposure to 2.78 ug/ml C60 for 3 h. **Mutations:** No increase after exposure to 100 ug/ml C60 for 8 passages (total exposure time 576 h, total exposure concentration 8 mg).	**ROS:** Increased after exposure to 2.78 ug/ml C60 for 3 h. **Mutations:** No increase after exposure to 100 ug/ml C60 for 8 passages (total exposure time 576 h, total exposure concentration 8 mg).

Table S5. Empirical evidence of the non-adjacent key event relationship between an increase in cellular ROS and chromosomal aberrations.

Citation	Toxicant	Model	ROS assay	Chromosomal aberration assay	Evidence Temporal
(Shih et al. 2021)	Arecoline	OEC-M1 cell line (oral squamous cell carcinoma)	Flow cytometer ROS Assay Kit 520 nm (Invitrogen, Cat. 88-5930)	Micronucleus assay	**ROS:** Increased after exposure to 100 ug/ml arecoline for 4 h.<br>**Chromosomal aberrations:** Increased after exposure to 100 ug/ml arecoline for 24 h.

Table S6. Quantitative evidence of the adjacent key event relationship between an increase in cellular ROS and oxidative DNA damage.

Citation	Toxicant	Model	ROS assay	Oxidative DNA damage assay	Description of the quantitative understanding
(Justiniano et al. 2017)	zinc pyrithione (ZnPT)	cultured malignant keratinocytes (SCC-25)	MitoSOX Red™ fluorescence assay	FPG-modified comet assay	Exposure to 5 µM ZnPT in SCC-25 keratinocytes, for 10 and 60 min, induced an increase in mitochondrial ROS (measured by the MitoSOX Red™ fluorescence assay) from 1 RFU to 1.5 RFU (10min) and 3.25 RFU (60min). Exposure to 5 µM ZnPT in SCC-25 keratinocytes, for 6 h induced increase in oxidative DNA damage (measured by the +Fpg comet assay) from a relative comet tail moment of 4 to 5.5.
(Gajski et al. 2015)	containinated water from abandoned uranimum mining area	Human blood (healthy female donor, age 24)	HPLC - MDA	FPG-modified comet assay	Exposure of human blood to contaminated water for 4 hours resulted in a significant increase in MDA, a marker of oxidative stress, from 10 nmol/g protein to 15 nmol/g protein. Exposure of human blood to contaminated water for 4 hours resulted in a significant increase in oxidative DNA damage (measured by the +Fpg comet assay) from 4% to 7% tail DNA.
(Gomez et al. 2009)	Methionine dietary restriction	Male Wistar Rat heart and liver mitochondria	homovanillic acid oxidation by H_2O_2	HPLC (8-oxodG)	Treatment with pyruvate, malate and methionine supplemented Male Wistar rats resulted in a significant increase of H_2O_2 in liver mitochondria from 0.05 nmol H_2O_2 /min mg protein to 0.1 nmol H_2O_2 /min mg protein. Treatment with pyruvate, malate and methionine supplemented Male Wistar rats resulted in a significant increase of 8-oxo-dG content in liver mitochondria from 3.5 8 OxodG/10^5 dG in mtDNA to 6.5 8 OxodG/10^5 dG in mtDNA.
(Inés Sanchez-Roman et al. 2012)	Methionine dietary restriction and aging	Male Wistar rat liver mitochondria	oxidation of homovanillic acid by H2O2	HPLC (8-oxodG and dG) for mt-DNA	Methionine restriction in the diet of male Wistar rats resulted in a significant decrease of liver mitochondrial H2O2 production from 0.13 nmol H2O2 /min mg protein to 0.06 nmol H2O2 /min mg protein. Methionine restriction in the diet of male Wistar rats resulted in a significant decrease of oxidative DNA damage in the liver mitochondria from 4.75 8-OxodG/10^5 dG in mtDNA to 3.5 8-OxodG/10^5 dG in mtDNA
(Taha et al. 2010)	iron-ascorbate (FE/ASC)-	Intestinal Caco-2/15 Cell Line mitochondria	HPLC - MDA	ELISA	Exposure of Caco-2/15 cells to 0.2 mM Fe and 2 mM ASC for 6 h resulted in a significant increase in mitochondrial MDA, a marker of oxidative stress, from 75 pmol/mg protein to 225 pmol/mg protein. Exposure of Caco-2/15 cells to 0.2 mM Fe and 2 mM ASC for 6 h resulted in a significant increase in mitochondrial 8-OHdG content from 0.2 ng/µg to 0.4 ng/µg.
(Ines Sanchez-Roman et al. 2011)	Methionine dietary restriction	Male Wistar rats, heart mitochondria	oxidation of homovanillic acid by H2O2	HPLC (8-oxodG and dG) for mt-DNA and ELISA for genomic DNA	Methionine restriction in the diet of male Wistar rats for 7 weeks resulted in a significant decrease in the rate or ROS production when treated with pyruvate, malate and rotenone, in heart mitochondria from 3.56 to 3.03 nmoles of H_2O_2/min. Methionine restriction in the diet of male Wistar rats for 7 weeks resulted in a significant decrease in 8-oxo-dG content from 6.5 8-oxo-dG/10^5 dG to 5.75 8-oxo-dG.
(Bruskov et al. 2002)	Heat	salmon sperm DNA	chemiluminescence in a peroxidase–luminol–p-iodophenol	ELISA	75°C heat for 4 hours was found to induce 2.4 nM H2O2 in phosphate buffer. 75°C hear for 24 hours was found in induce 96 8-oxodG/10^5 dG in salmon sperm DNA

(Lajmanovich et al. 2015)	Pesticide 2,4-D	adult male of R. arenarum (toad species)	GST activity	Endo III and FPG-modified comet assays	Dermal exposure of male R. arenarum (toads) to 20mg/L 2,4-D for 48, resulted in significantly increased blood glutathione S-transferase (GST) activity measured. Dermal exposure of male R. arenarum (toads) to 20 mg/L 2,4-D for 48 h, resulted in no significant change to oxidative DNA damage in the blood.
(Soberanes et al. 2012)	PM2.5	male-C57BL/6 mice	mitochondrial matrix localized oxidant-sensitive ratiometric probe (mito-Ro-GFP)	8-oxo-dG ELISA	Exposure of mouse lung epithelial cells to PM for 48 hours resulted in a significant increase in oxidation of the mitochondrial ROS probe mito-Ro-GFP from 5% to 10% (5 µg/cm2 PM) and 40% (10 µg/cm^2 PM). Exposure of mouse lung epithelial cells to concentrated PM for 72 hours resulted in a significant increase in 8-oxodG positve nuclei from 2 positive nuclei/field to 50 positive nuclei/field.
(Vadrot et al. 2012)	Tumour necrosis factor alpha	human hepatoma HepG2 cells	Luminol-amplified chemiluminescence (LAC)	ELISA	Exposure of HepG2 cells to 30ng/ml TNF-alpha for resulted in a a peak of ROS chemilunesence at 10 min, a significant increase of ROS to 170% of the basal chemiluminesence. Exposure of HepG2 cells to 30 ng/ml TNF-alpha for 15 min, 30 min, 1 h and 3h resulted in a peak increase in 8-oxo-dG at 1 h, from 0.3 8-oxo-dG ng/mg DNA to 1 8-oxo-dG ng/mg DNA.
(Espinosa et al. 2007)	hypertension	human subjects with hypertension	HPLC-EC determination of GSH (reduced glutathione) and GSSG (oxidzed glutathione) content	hPLC-EC (8-Oxo-dG and dG)	Blood samples from humans with hypertension had significantly increased oxidized glutathione to reduced glutathione ratios (GSSG nmol mg protein-1 / GSH nmol mg protein-1) from 0.60 to 5.80. Blood samples from humans with hypertension had significantly increased mitochondrial 8-oxo-dG content from 3.97 8-oxo-dG(nmol)/creatine(mmol) to 5.94 8-oxo-dG(nmol)/creatine(mmol) and nuclear 8-oxo-dG content from 5.40 8-oxo-dG(nmol)/creatine(mmol) to 6.65 8-oxo-dG(nmol)/creatine(mmol).
(Mustafa et al. 2011)	hypoxia (hyperoxic and hypoxic conditions)	common carp, Cyprinus carpio L	Glutathione peroxidase (GPx) activity	FPG-modified comet assay	Exposure of carp to hyperoxic conditions for 30 days resulted in an increase in GPx activity from 8*10^2 GPx activity nmol/min/ml to 1.1*10^2 GPx activity nmol/min/ml. Exposure of carp to hyperoxic conditions for 30 days resulted in an increase in % tail DNA in the FPG modified cometchip assay from 30% to 45%.

Supplementary References

Alsharif, Naser Z, and Ezdihar A Hassoun. 2004. “Thymic Atrophy , Production of Reactive Oxygen Species and DNA Damage in C57BL / 6J Mice.” *Basic & clinical pharmacology & toxicology* 95: 131–38.

Babbar, Naveen, and Robert A Casero. 2006. “Tumor Necrosis Factor-Alpha Increases Reactive Oxygen Species by Inducing Spermine Oxidase in Human Lung Epithelial Cells: A Potential Mechanism for Inflammation-Induced Carcinogenesis.” *Cancer research (Chicago, Ill.)* 66(23): 11125–30.

Beattie, Matthew C. et al. 2013. “Aging and Luteinizing Hormone Effects on Reactive Oxygen Species Production and DNA Damage in Rat Leydig Cells.” *Biology of Reproduction* 88(4): 1–7.

Brar, Sukhdev S. et al. 2012. “Mitochondrial DNA-Depleted A549 Cells Are Resistant to Bleomycin.” *American Journal of Physiology - Lung Cellular and Molecular Physiology* 303(5): 413–24.

Bruskov, Vadim I., Lyudmila V. Malakhova, Zhaksylyk K. Masalimov, and Anatoly V. Chernikov. 2002. “Heat-Induced Formation of Reactive Oxygen Species and 8-Oxoguanine, a Biomarker of Damage to DNA.” *Nucleic Acids Research* 30(6): 1354–63.

Cai, Xiaoqiang, Nina Pan, and Guolin Zou. 2007. “Copper-1,10-Phenanthroline-Induced Apoptosis in Liver Carcinoma Bel-7402 Cells Associates with Copper Overload, Reactive Oxygen Species Production, Glutathione Depletion and Oxidative DNA Damage.” *BioMetals* 20(1): 1–11.

Chan, Tze Khee, W. S. Daniel Tan, Hong Yong Peh, and W. S. Fred Wong. 2017. “Aeroallergens Induce Reactive Oxygen Species Production and DNA Damage and Dampen Antioxidant Responses in Bronchial Epithelial Cells.” *The Journal of Immunology* 199(1): 39–47.

Chen, Chen et al. 2020. “Ionomycin-Induced Mouse Oocyte Activation Can Disrupt Preimplantation Embryo Development through Increased Reactive Oxygen Species Reaction and DNA Damage.” *Molecular human reproduction* 26(10): 773–83.

Espinosa, Olga et al. 2007. “Urinary 8-Oxo-7, 8-Dihydro-2′-Deoxyguanosine (8-Oxo-DG), a Reliable Oxidative Stress Marker in Hypertension.” *Free Radical Research* 41(5): 546–54.

Flaherty, Renee L et al. 2017. “Glucocorticoids Induce Production of Reactive Oxygen Species/Reactive Nitrogen Species and DNA Damage through an INOS Mediated Pathway in Breast Cancer.” *Breast cancer research : BCR* 19(1): 35.

Froeling, Fieke E M et al. 2019. “Bioactivation of Napabucasin Triggers Reactive Oxygen Species-Mediated Cancer Cell Death.” *Clinical cancer research* 25(23): 7162–74.

Gagnaire, Béatrice et al. 2020. “Tritiated Water Exposure in Zebrafish (Danio Rerio): Effects on the Early-Life Stages.” *Environmental Toxicology and Chemistry* 39(3): 648–58. http://dx.doi.org/10.1002/etc.4650.

Gajski, Goran et al. 2015. “Toxicity Assessment of the Water Used for Human Consumption from the Cameron/Tuba City Abandoned Uranium Mining Area Prior/after the Combined Electrochemical Treatment/Advanced Oxidation.” *Environmental Science and Pollution Research* 22(1): 516–26.

Gil, Ha Na et al. 2019. “A Synthetic Chalcone Derivative, 2-Hydroxy-3′,5,5′-Trimethoxychalcone (DK-139), Triggers Reactive Oxygen Species-Induced Apoptosis Independently of P53 in A549 Lung Cancer Cells.” *Chemico-biological interactions* 298: 72–79.

Gomez, Jose et al. 2009. “Effect of Methionine Dietary Supplementation on Mitochondrial Oxygen Radical Generation and Oxidative DNA Damage in Rat Liver and Heart.” *Journal of Bioenergetics and Biomembranes* 41(3): 309–21.

Greenshields, Anna L, Trevor G Shepherd, and David W Hoskin. 2017. “Contribution of Reactive Oxygen Species to Ovarian Cancer Cell Growth Arrest and Killing by the Anti-malarial Drug Artesunate.” *Molecular carcinogenesis* 56(1): 75–93.

Gu, Mei mei et al. 2017. “Molecular Characterization and Function of the Prohibitin2 Gene in Litopenaeus Vannamei Responses to Vibrio Alginolyticus.” *Developmental and Comparative Immunology* 67: 177–88. http://dx.doi.org/10.1016/j.dci.2016.10.004.

Itziou, A., M. Kaloyianni, and V. K. Dimitriadis. 2011. “In Vivo and in Vitro Effects of Metals in Reactive Oxygen Species Production, Protein Carbonylation, and DNA Damage in Land Snails Eobania Vermiculata.” *Archives of Environmental Contamination and Toxicology* 60(4): 697–707.

De Iuliis, Geoffry N., Rhiannon J. Newey, Bruce V. King, and R. John Aitken. 2009. “Mobile Phone Radiation Induces Reactive Oxygen Species Production and DNA Damage in Human Spermatozoa in Vitro.” *PLoS ONE* 4(7).

Jacobsen, Nicklas Raun et al. 2008. “Genotoxicity, Cytotoxicity, and Reactive Oxygen Species Induced by Single-Walled Carbon Nanotubes and C60 Fullerenes in the FE1-Muta™Mouse Lung Epithelial Cells.” *Environmental and molecular mutagenesis* 49(6): 476–87.

Joseph, P., N. N. Bhat, D. Copplestone, and Y. Narayana. 2014. “Production of Gamma Induced Reactive Oxygen Species and Damage of DNA Molecule in HaCaT Cells under Euoxic and Hypoxic Condition.” *Journal of Radioanalytical and Nuclear Chemistry* 302(2): 983–88.

Justiniano, Rebecca et al. 2017. “A Topical Zinc Ionophore Blocks Tumorigenic Progression in UV-Exposed SKH-1 High-Risk Mouse Skin.” *Photochemistry and Photobiology* 93(6): 1472–82.

Lajmanovich, Rafael C. et al. 2015. “Harmful Effects of the Dermal Intake of Commercial Formulations Containing Chlorpyrifos, 2,4-D, and Glyphosate on the Common Toad Rhinella Arenarum (Anura: Bufonidae).” *Water, Air, and Soil Pollution* 226(12).

Lee, B, J S Hwang, and D G Lee. 2020. “Antibacterial Action of Lactoferricin B like Peptide against Escherichia Coli: Reactive Oxygen Species-induced Apoptosis-like Death.” *Journal of applied microbiology* 129(2): 287–95.

Lee, Kwanghyun et al. 2015. “Cyclo(Phenylalanine-proline) Induces DNA Damage in Mammalian Cells via Reactive Oxygen Species.” *Journal of*

cellular and molecular medicine 19(12): 2851–64.

Li, Qianqian, Shuangqing Peng, Zhiguo Sheng, and Yimei Wang. 2010. "Ofloxacin Induces Oxidative Damage to Joint Chondrocytes of Juvenile Rabbits: Excessive Production of Reactive Oxygen Species, Lipid Peroxidation and DNA Damage." *European Journal of Pharmacology* 626(2–3): 146–53. http://dx.doi.org/10.1016/j.ejphar.2009.09.044.

Luukkonen, Jukka, Jukka Juutilainen, and Jonne Naarala. 2010. "Combined Effects of 872 MHz Radiofrequency Radiation and Ferrous Chloride on Reactive Oxygen Species Production and DNA Damage in Human SH-SY5Y Neuroblastoma Cells." *Bioelectromagnetics* 31(6): 417–24.

Mittal, Sandeep, and Alok K. Pandey. 2014. "Cerium Oxide Nanoparticles Induced Toxicity in Human Lung Cells: Role of ROS Mediated DNA Damage and Apoptosis." *BioMed Research International* 2014.

Mustafa, Sanaa A., Sherain N. Al-Subiai, Simon J. Davies, and Awadhesh N. Jha. 2011. "Hypoxia-Induced Oxidative DNA Damage Links with Higher Level Biological Effects Including Specific Growth Rate in Common Carp, Cyprinus Carpio L." *Ecotoxicology* 20(6): 1455–66.

Ni, Chien-Hang et al. 2014. "Chrysophanol-Induced Cell Death (Necrosis) in Human Lung Cancer A549 Cells Is Mediated through Increasing Reactive Oxygen Species and Decreasing the Level of Mitochondrial Membrane Potential." *Environmental toxicology* 29(7): 740–49.

Nie, Zheng-Wen et al. 2019. "Thiamethoxam Inhibits Blastocyst Expansion and Hatching via Reactive-Oxygen Species–Induced G2 Checkpoint Activation in Pigs." *Cellular signalling* 53: 294–303.

Pagoria, Dustin, Abert Lee, and Werner Geurtsen. 2005. "The Effect of Camphorquinone (CQ) and CQ-Related Photosensitizers on the Generation of Reactive Oxygen Species and the Production of Oxidative DNA Damage." *Biomaterials* 26(19): 4091–99.

Pan, Min Hsiung et al. 2007. "5-Hydroxy-3,6,7,8,3′,4′-Hexamethoxyflavone Induces Apoptosis through Reactive Oxygen Species Production, Growth Arrest and DNA Damage-Inducible Gene 153 Expression, and Caspase Activation in Human Leukemia Cells." *Journal of Agricultural and Food Chemistry* 55(13): 5081–91.

Preston, Thomas J, Jeffrey T Henderson, Gordon P McCallum, and Peter G Wells. 2009. "Base Excision Repair of Reactive Oxygen Species–Initiated 7,8-Dihydro-8-Oxo-2′-Deoxyguanosine Inhibits the Cytotoxicity of Platinum Anticancer Drugs." *Molecular cancer therapeutics* 8(7): 2015–26.

Ren, Guowen et al. 2015. "Cucurbitacin B Induces DNA Damage and Autophagy Mediated by Reactive Oxygen Species (ROS) in MCF-7 Breast Cancer Cells." *Journal of natural medicines* 69(4): 522–30.

———. 2016. "Psoralidin Induced Reactive Oxygen Species (ROS)-Dependent DNA Damage and Protective Autophagy Mediated by NOX4 in Breast Cancer Cells." *Phytomedicine (Stuttgart)* 23(9): 939–47.

Russo, A et al. 2006. "Propolis Protects Human Spermatozoa from DNA Damage Caused by Benzo[a]Pyrene and Exogenous Reactive Oxygen

Species." *Life sciences (1973)* 78(13): 1401–6.

Samara, Aladin et al. 2021. "Deferasirox Induces Cyclin D1 Degradation and Apoptosis in Mantle Cell Lymphoma in a Reactive Oxygen Species- and GSK3β-dependent Mechanism." *British journal of haematology* 192(4): 747–60.

Sanchez-Roman, Ines et al. 2011. "Forty Percent Methionine Restriction Lowers DNAmethylation, Complex i ROS Generation, and Oxidative Damage to MtDNA and Mitochondrial Proteins in Rat Heart." *Journal of Bioenergetics and Biomembranes* 43(6): 699–708.

Sanchez-Roman, Inés et al. 2012. "Effects of Aging and Methionine Restriction Applied at Old Age on ROS Generation and Oxidative Damage in Rat Liver Mitochondria." *Biogerontology* 13(4): 399–411.

Shih, Yin Hwa et al. 2021. "Effects of Melatonin to Arecoline-Induced Reactive Oxygen Species Production and DNA Damage in Oral Squamous Cell Carcinoma." *Journal of the Formosan Medical Association* 120(1): 668–78. https://doi.org/10.1016/j.jfma.2020.07.037.

Soberanes, Saul et al. 2012. "Particulate Matter Air Pollution Induces Hypermethylation of the P16 Promoter Via a Mitochondrial ROS-JNK-DNMT1 Pathway." *Scientific Reports* 2: 1–8.

Taha, Rame et al. 2010. "Oxidative Stress and Mitochondrial Functions in the Intestinal Caco-2/15 Cell Line." *PLoS ONE* 5(7): 1–10.

Toduka, Yousuke, Tatsushi Toyooka, and Yuko Ibuki. 2012. "Flow Cytometric Evaluation of Nanoparticles Using Side-Scattered Light and Reactive Oxygen Species-Mediated Fluorescence–Correlation with Genotoxicity." *Environmental science & technology* 46(14): 7629–36.

Vadrot, Nathalie et al. 2012. "Mitochondrial DNA Maintenance Is Regulated in Human Hepatoma Cells by Glycogen Synthase Kinase 3β and P53 in Response to Tumor Necrosis Factor α." *PLoS ONE* 7(7): 9–14.

Wang, Enyin, Yan Huang, Qingyun Du, and Yingpu Sun. 2017. "Silver Nanoparticle Induced Toxicity to Human Sperm by Increasing ROS(Reactive Oxygen Species) Production and DNA Damage." *Environmental Toxicology and Pharmacology* 52(November 2016): 193–99. http://dx.doi.org/10.1016/j.etap.2017.04.010.

Wang, Zhixiong et al. 2021. "Photoacoustic Cavitation-Ignited Reactive Oxygen Species to Amplify Peroxynitrite Burst by Photosensitization-Free Polymeric Nanocapsules." *Angewandte Chemie (International ed.)* 60(9): 4720–31.

Weyemi, U. et al. 2012. "ROS-Generating NADPH Oxidase NOX4 Is a Critical Mediator in Oncogenic H-Ras-Induced DNA Damage and Subsequent Senescence." *Oncogene* 31(9): 1117–29.

Yin, Y. et al. 2019. "Arsenic Enhances Cell Death and DNA Damage Induced by Ultraviolet B Exposure in Mouse Epidermal Cells through the Production of Reactive Oxygen Species." *Clinical and Experimental Dermatology* 44(5): 512–19.

Zhang, J. et al. 2015. "Aflatoxin B1 and Aflatoxin M1 Induced Cytotoxicity and DNA Damage in Differentiated and Undifferentiated Caco-2 Cells." *Food and Chemical Toxicology* 83: 54–60.
